# Evaluation of Anesthesia Management During Peroral Endoscopic Myotomy in Patients with Achalasia: A Retrospective Study

**DOI:** 10.3390/jcm14186504

**Published:** 2025-09-16

**Authors:** Mukadder Sanli, Sami Akbulut, Muharrem Ucar, Yilmaz Bilgic

**Affiliations:** 1 Department of Anesthesiology and Reanimation, Inonu University Faculty of Medicine, 44280 Malatya, Turkey; 2 Department of Surgery, Inonu University Faculty of Medicine, 44280 Malatya, Turkey; 3 Liver Transplant Institute, Inonu University Faculty of Medicine, 44280 Malatya, Turkey; 4 Department of Gastroenterology, Inonu University Faculty of Medicine, 44280 Malatya, Turkey

**Keywords:** peroral endoscopic myotomy, achalasia, anesthesia management, rapid sequence induction, carbon dioxide insufflation, aspiration risk, perioperative complications

## Abstract

**Background**: Achalasia is a primary esophageal motility disorder characterized by impaired relaxation of the lower esophageal sphincter (LES) and absent peristalsis, which increases the risk of aspiration during anesthesia. Peroral endoscopic myotomy (POEM) is a minimally invasive therapeutic approach requiring tailored anesthetic management. This study aimed to evaluate perioperative anesthesia management during POEM, focusing on ventilation parameters, intraoperative hemodynamics, laboratory changes, and the incidence and severity of postoperative complications. **Methods**: A retrospective analysis was conducted on 51 patients who underwent POEM between June 2016 and April 2025. Demographic features, anesthesia techniques, intraoperative physiologic parameters, hematologic profiles, and postoperative complications were evaluated. Standard preoperative fasting protocols were implemented. Rapid sequence induction (RSI) with propofol and rocuronium was followed by endotracheal intubation. Desflurane was used for maintenance anesthesia, with ventilation settings adjusted to limit end-tidal carbon dioxide (ETCO_2_) elevation. **Results:** The median age of patients was 48 years, with a slight female (52.9%) predominance. Most patients were American Society of Anesthesiologists (ASA) II (64.7%) or ASA III (35.3%) scores and had comorbid hypertension (31.4%) or diabetes (11.8%). The median anesthesia duration was 180 min, and the peak inspiratory pressure remained stable at 25 mmHg. Oxygen saturation (SpO_2_) improved during the procedure, while ETCO_2_ increased from baseline to 49 mmHg by the end. Blood pressure declined transiently but recovered intraoperatively. Hematologic analysis showed significant increases in white blood cell (WBC) and neutrophils and mild decreases in hemoglobin, hematocrit, and platelets. Early postoperative complications included subcutaneous emphysema (19.6%), minor bleeding (9.8%), and pneumoperitoneum (7.84%). Two patients required tube thoracostomy due to pneumothorax, but no patient developed a complication requiring surgical exploration. During a median follow-up of 546 days, no mortality was reported. Long-term complications were infrequent, with gastroesophageal reflux disease (GERD) (3.92%) and esophagitis (1.96%) being the most notable. **Conclusions:** POEM can be performed safely with appropriate anesthetic management. Despite significant physiologic changes during carbon dioxide (CO_2_) insufflation, no life-threatening complications occurred, and the majority of adverse events were minor and self-limiting. Close intraoperative monitoring and interdisciplinary coordination contribute to favorable perioperative outcomes.

## 1. Introduction

Achalasia is a primary esophageal motility disorder characterized by impaired relaxation of the lower esophageal sphincter (LES) and the absence of normal esophageal peristalsis [1]. Achalasia was first described by Thomas Willis in 1674. The term ‘achalasia’ was later coined by Hurst in 1927, who suggested that the disorder might result from absent relaxation of the sphincter due to changes in Auerbach’s plexus [2]. It is rare, with an estimated incidence of 1.0–3.1 per 100,000 individuals annually and a prevalence of approximately 10–45 per 100,000, affecting both genders equally, although some studies suggest a slight female predominance, and it is most often diagnosed between 20 and 50 years of age [1,3,4,5].

The pathophysiology of achalasia involves the progressive loss of ganglion cells in the myenteric (Auerbach’s) plexus, particularly those responsible for inhibitory neurotransmission mediated by nitric oxide and vasoactive intestinal peptide. This neuronal degeneration leads to an imbalance between excitatory and inhibitory signals, resulting in sustained contraction of the LES and loss of coordinated peristalsis in the esophageal body. Histopathological studies frequently reveal chronic inflammation, fibrosis, and lymphocytic infiltration around the myenteric plexus, suggesting possible autoimmune or viral etiologies [2,6,7,8,9].

Achalasia is categorized into various groups based on anatomical features, physiological characteristics, and underlying causes. Achalasia can be classified into two main categories: primary (idiopathic) and secondary forms [10,11]. Primary achalasia arises from degeneration of inhibitory neurons in the myenteric plexus without a known underlying cause, whereas secondary achalasia, also referred to as pseudoachalasia, is typically caused by malignancies or other external factors leading to mechanical or functional obstruction at the gastroesophageal junction (GEJ). Furthermore, based on high-resolution manometry (HRM) findings, achalasia is subtyped according to the Chicago Classification into three types: Type I (classic achalasia), characterized by minimal esophageal pressurization, Type II, featuring panesophageal pressurization, and Type III, involving spastic contractions in the distal esophagus [10,12]. This subclassification has significant implications for treatment planning and prognosis.

Typical symptoms of achalasia include difficulty swallowing known as dysphagia, retrosternal chest pain, regurgitation which refers to the return of undigested food or esophageal contents into the mouth, unintentional weight loss, and esophageal dilation accompanied by marked narrowing at the GEJ [6,7]. Dysphagia is the most prevalent and characteristic symptom, affecting approximately 70–97% of patients [6,13]. It typically begins with solids and often progresses to include liquids over time [14]. Regurgitation frequently occurs postprandially or in the recumbent position, is usually non-acidic and painless, and may involve undigested food [15]. Chest pain can be meal-related or spontaneous [15,16]. Due to the LES’s failure to relax properly and the lack of peristalsis, swallowed food and liquids cannot pass effectively into the stomach. They accumulate above the dysfunctional sphincter. This chronic obstruction and increased pressure lead to stretching of the esophageal wall. Over time, this results in progressive dilation of the esophagus (megaesophagus), a hallmark of advanced disease visible radiographically [17]. This dilation further exacerbates the risk of food stasis and fermentation.

Diagnosis of achalasia is based on a combination of clinical symptoms, imaging, esophagogastroduodenoscopy (EGD), and HRM, which remains the gold standard [18,19,20,21]. Manometry confirms absent peristalsis and impaired LES relaxation, enabling classification according to the Chicago Classification into Types I, II, and III, which have implications for treatment planning. Barium swallowing often shows the classic bird’s beak appearance, and endoscopy helps exclude malignancy causing pseudoachalasia [5,22]. Emerging technologies like Functional Lumen Imaging Probe (FLIP) further aid in diagnosis [22,23].

The treatment of achalasia focuses on relieving the functional obstruction at the lower esophageal sphincter and improving esophageal emptying [14,24]. Options include pneumatic balloon dilation, laparoscopic or robotic Heller myotomy, botulinum toxin injection, and pharmacological therapies like nitrates or calcium channel blockers, though medications are generally less effective and reserved for those unfit for invasive procedures [25,26,27]. Pneumatic dilation and Heller myotomy have long been established as effective interventions [28]. Botulinum toxin provides temporary relief and is used in patients who are poor surgical candidates [29].

Peroral endoscopic myotomy (POEM), first described by Inoue et al. [30], is a minimally invasive endoscopic surgical technique developed for the treatment of esophageal achalasia and other esophageal motility disorders [31]. It involves creating a mucosal incision, performing submucosal tunneling, and cutting the circular muscle fibers across the GEJ, followed by closure of the mucosal entry with endoscopic clips [32,33]. POEM has shown comparable efficacy to surgical myotomy and is particularly beneficial in patients with Type III achalasia, who have spastic contractions of the distal esophagus [1,34]. Although technically demanding, POEM is considered safe when performed by experienced operators [35].

As the first endoscopic procedure requiring general anesthesia, POEM poses significant challenges for anesthesia management [33,36,37,38,39]. These challenges include patient positioning, prolonged duration of endoscopic procedure, increased intra-abdominal pressure due to pneumoperitoneum, and potential complications due to carbon dioxide (CO_2_) leakage into surrounding tissues [38]. Overall, POEM has a reported complication rate ranging broadly from 7% to as high as 55%, with many of these complications categorized as insufflation-related adverse events, depending on patient factors and procedural expertise [36]. The most common complications are subcutaneous emphysema, pneumothorax, pneumomediastinum, pneumoperitoneum, and pleural effusion, which are typically mild and manageable [33,36,38,40,41]. Rare but severe complications, such as mediastinitis, esophageal perforation, or significant bleeding, occur in less than 1% of cases [35,38].

Given these procedure-related challenges, managing anesthesia in patients with achalasia undergoing POEM requires a careful approach, focusing on perioperative safety and individualized strategies. Recent studies have reported fasting times ranging from 8 to 48 h. The majority recommend a clear liquid diet for at least 24 h or a low-residue diet for 48 h prior to the procedure, particularly for patients at high risk of esophageal retention [33,37,39]. Preprocedural esophageal emptying using endoscopy may be beneficial, especially in patients with significant residual food content [42,43]. The primary concern remains the risk of aspiration due to large volumes of retained food and fluid in the dilated esophagus, particularly during induction of anesthesia when protective airway reflexes are lost [33,36,37,38,39]. Such aspiration can lead to severe pneumonia and other pulmonary infections [42]. Additionally, the dilated esophagus can distort normal anatomy, potentially complicating endotracheal intubation. Therefore, meticulous preoperative evaluation, prolonged fasting protocols, possible esophageal clearance procedures, and strict aspiration prophylaxis—including rapid sequence induction (RSI) and cricoid pressure—are critical for safe anesthetic management [44]. Semi-reclined positioning during induction and intubation is recommended to reduce aspiration risk [37]. Furthermore, the recent literature suggests the use of hyperventilation and percutaneous needle decompression to manage elevated CO_2_ levels during the procedure [36,38].

In addition to anesthetic management, outcomes such as symptom relief, recurrence rates, and postoperative gastroesophageal reflux disease (GERD) are critical aspects in evaluating the overall success of POEM [45]. Recent studies indicate that POEM achieves symptom relief rates about 90%, though postoperative GERD occurs in approximately 19–50% of patients, highlighting the importance of balancing procedural efficacy with overall patient health and long-term health-related quality of life (HRQOL) [45,46]. This study aims to evaluate both the anesthetic considerations and the endoscopic and long-term clinical outcomes associated with POEM, providing a comprehensive analysis that integrates perioperative management with patient-centered results, in alignment with current evidence-based practices.

## 2. Material and Methods

### 2.1. Study Design and Patient Population

This retrospective study focused on 51 patients who underwent POEM under general anesthesia due to achalasia between June 2016 and April 2025 at the Department of Gastroenterology, Inonu University Faculty of Medicine. Data were retrieved from the hospital patient information and management system (HBYS—Hospital Information Management System) and the standard patient record forms used in the Department of Anesthesiology, including demographic, biochemical, and clinical variables of these patients, which were analyzed.

### 2.2. Definition of Achalasia

In our center, the diagnosis of achalasia was established by evaluating the clinical presentation, radiological imaging, endoscopic examination, and HRM findings together. The criteria included (i) typical symptoms such as progressive dysphagia (for solids and liquids), regurgitation, chest pain, and/or weight loss; (ii) timed barium esophagogram showing a bird-beak appearance at the GEJ and proximal esophageal dilation, consistent with achalasia (esophageal caliber was considered normal if ≤4 cm, mildly dilated if 4.1–6 cm, and markedly dilated if >6 cm or if the distal esophagus had a sigmoid configuration); (iii) endoscopic observation of a tightly closed LES without mucosal abnormalities or evidence of mechanical obstruction; (iv) HRM showing elevated integrated relaxation pressure (IRP > 15 mmHg) and absence of peristalsis, with subtype classification into Type I, II, or III [47,48,49].

### 2.3. Preoperative Preparation and Anesthesia Protocol

Patients fasted for 24–36 h prior to surgery. Esophageal clearance with EGD was performed in 30 cases before transfer to the operating room. No premedication was administered. Upon arrival, routine monitoring (ECG, SpO_2_, noninvasive blood pressure, and capnography) was instituted, and radial artery cannulation was performed in five patients for invasive blood pressure monitoring and arterial blood gas analysis. Induction was achieved with propofol 2–3 mg/kg, lidocaine 1 mg/kg, fentanyl 1 mcg/kg, and rocuronium 1.2 mg/kg. RSI was performed in a semi-sitting position using the Sellick maneuver with a spiral endotracheal tube to minimize aspiration risk; cricoid pressure was released after confirmation of intubation. Maintenance anesthesia was provided with desflurane inhalation at a target MAC of 1.0 in a 50% air–oxygen mixture. Ventilation was initially set to volume-controlled mode, with adjustments in respiratory rate and minute ventilation to maintain ETCO_2_ < 45 mmHg [36,50,51]. If ETCO_2_ exceeded this threshold, ventilation was increased and the procedure paused when necessary. When PIP rise above 25 cm H_2_O, ventilation was switched to pressure-controlled mode. During POEM, PIP frequently increased due to gastric distension or pneumoperitoneum; in such cases, tidal volume was reduced, respiratory rate increased, and the abdomen examined. If pressures remained elevated, CO_2_ insufflation was reduced, gastric suction applied, and percutaneous needle decompression performed. In line with our institutional practice and literature recommendations, PIP was maintained <30 cm H_2_O to avoid barotrauma [36]. Throughout induction and maintenance, only two patients required inotropic support, and this was necessary for a very short duration.

### 2.4. POEM Procedure

All procedures were performed under general anesthesia with prophylactic administration of 30 mg/kg (maximum 2000 mg) intravenous cefazolin sodium at induction [52,53]. Patients were placed in the supine position with both arms adducted and the head rotated 30 degrees to the left, toward the endoscopist. The oral cavity was aspirated prior to scope insertion. CO_2_ insufflation was used throughout the procedure to improve visualization and facilitate submucosal dissection, with the flow rate maintained at approximately 1.2 L/min. Capnography was continuously monitored, and increases in ETCO_2_ were managed by adjusting minute ventilation. The POEM procedure was conducted in four sequential steps: (i) mucosal entry (mucosotomy), (ii) submucosal tunneling, (iii) myotomy, and (iv) mucosal closure [49]. Mucosal entry was typically initiated 10–15 cm proximal to the GEJ [49,54]. After a submucosal cushion was created with a saline and blue dye mixture, a 2 cm mucosal incision was made to access the submucosal plane [49]. A tunnel was developed using alternating injections and energy dissection, extending approximately 2–3 cm beyond the GEJ to ensure complete division of the lower esophageal sphincter [49,55]. During submucosal dissection, larger blood vessels were individually coagulated to prevent bleeding. Myotomy began 3–5 cm distal to the site of mucosotomy, targeting primarily the circular muscle fibers while preserving the longitudinal layer when feasible [49,56]. Although the optimal depth of myotomy remains debated, selective myotomy is generally preferred to avoid entering mediastinal planes. Some cases in the literature have shown that full-thickness myotomy can reduce operative time without compromising safety. At the completion of the myotomy, the tunnel and mucosa were carefully inspected to detect any inadvertent perforation or mucosal defect [49]. The mucosal entry site was then closed using endoscopic clips. Following the procedure, neuromuscular blockade was reversed with intravenous sugammadex (4 mg/kg), and patients were extubated and transferred to the intensive care unit (ICU) for monitoring. All patients were observed in the ICU for approximately 24 h, followed by an average of 2 days in the surgical ward. Postoperative pain management included intravenous tramadol (1 mg/kg) and paracetamol (1000 mg) [57]. No major complications were encountered, and all patients maintained stable vital signs during the perioperative and postoperative periods.

### 2.5. Collected Variables and Outcome Measures

The study parameters included age, gender, comorbidities (such as hypertension, diabetes mellitus, cancer, and chronic pulmonary diseases), American Society of Anesthesiologists status (ASA) scores, and biochemical analyses (measured both preoperatively and before discharge). Additional data included anesthetic agents used, duration of the POEM procedure and anesthesia, postoperative analgesic protocols, ICU and hospital stay durations, and follow-up period. Intraoperative parameters measured during POEM included ETCO_2_, oxygen saturation (SpO_2_), systolic and diastolic blood pressure, mean arterial pressure (MAP), and PIP, recorded at baseline, at 10 min, and at the end of the procedure. Baseline LES and upper esophageal sphincter (UES) pressures (mmHg) were measured preoperatively. The distance from the incisors to the LES was also recorded during endoscopic evaluation. In addition, POEM-related morbidity and mortality, technical and clinical success rates, recurrence of achalasia, and the incidence of GERD and esophagitis were assessed as primary clinical outcomes.

### 2.6. Study Protocol and Ethics Committee Approval

This retrospective study involving human participants was conducted in accordance with the ethical standards of the institutional and national research committees and with the 1964 Helsinki Declaration and its later amendments or comparable ethical standards. Ethical approval for this non-interventional study was obtained from the Inonu University Institutional Review Board (IRB) under protocol number 7899, dated 7 May 2025. All participants provided both verbal and written informed consent prior to undergoing the POEM procedure. The STROBE (Strengthening the Reporting of Observational Studies in Epidemiology) guidelines were followed to enhance reporting transparency and reduce potential bias in this study [58].

### 2.7. Biostatistical Analysis

The statistical analysis for this study was performed using IBM SPSS Statistics version 25.0 (IBM Corp., Armonk, NY, USA). Descriptive statistics—including means, standard deviations, and percentile values—were used to summarize the data. Continuous variables are presented as medians with interquartile ranges (IQR: Q1–Q3) and 95% confidence intervals (95% CI: lower–upper limits). These statistical measures were used to identify clinically meaningful thresholds and assess variability in physiological parameters during anesthesia management in POEM procedures. For repeated measurements involving two time points, the non-parametric Wilcoxon signed-rank test was used to assess differences between preoperative and postoperative values. For variables measured at three or more time points, the Friedman two-way analysis of variance (ANOVA) test was applied to evaluate overall changes across the perioperative period. Statistical significance was set at a two-tailed *p*-value of < 0.05.

## 3. Results

A detailed overview of the study results is provided in Table 1, Table 2 and Table 3. Table 1 summarizes the demographic and clinical profiles of the study population, including comorbidities and postoperative complications. Table 2 presents intraoperative physiological and hemodynamic parameters recorded at various time points, while Table 3 highlights hematologic changes before and after the procedure, reflecting the systemic response induced by POEM.

A total of 51 patients underwent POEM during the study period. The median age was 48 years (IQR: 40–63), and there was a slight predominance of female patients (52.9%). The most common blood group was 0 Rh(+) (45.1%), followed by B Rh(+) (27.5%), A Rh(+) (23.5%), and AB Rh(+) (3.9%). The median height and weight were 164 cm (IQR: 160–173) and 55 kg (IQR: 50–63), respectively. Regarding preoperative risk status, 64.7% of patients were classified as ASA II, while 35.3% were ASA III. Hypertension and diabetes mellitus were the most frequent comorbidities, affecting 31.4% and 11.8% of the POEM patients, respectively. These findings suggest a predominance of moderate-risk patients with manageable systemic disease undergoing POEM (Table 1).

In terms of intraoperative parameters, the median duration of anesthesia was 180 min (IQR: 165–185), indicating that POEM is a time-consuming but stable endoscopic procedure. The median PIP was 25 mmHg (IQR: 25–26), showing no evidence of excessive airway pressure during ventilation. SpO_2_ improved from a median baseline value of 95% (IQR: 94–96) to 98% (IQR: 95–99) by the end of the procedure. Meanwhile, ETCO_2_ levels rose from 34 mmHg (IQR: 32–38) to 49 mmHg (IQR: 45–52), reflecting expected physiological changes during insufflation and pneumodissection (Table 2).

Blood pressure showed transient intraoperative hypotension: systolic BP declined from a median of 124 mmHg (IQR: 115–133) to 96 mmHg (IQR: 88–109) at 10 min, then partially recovered to 108 mmHg (IQR: 97–119). Diastolic and mean arterial pressures (MAP) followed similar dynamics, with MAP decreasing from 88 mmHg (IQR: 81–96) to 71 mmHg (IQR: 65–78), then recovering to 76 mmHg (IQR: 70–86). These fluctuations remained within safe anesthetic limits and were managed without pharmacologic intervention (Table 2).

Significant changes were observed in physiological parameters measured at three different perioperative time points. According to the Friedman two-way ANOVA test, SpO_2_, ETCO_2_, systolic blood pressure, diastolic blood pressure, and MAP all demonstrated statistically significant differences across the measurement periods (*p* < 0.001). Post hoc pairwise comparisons revealed that: (i) SpO_2_ levels increased significantly from baseline to the 10th minute and at the end of the procedure (*p* < 0.001 for both). (ii) ETCO_2_ levels exhibited a stepwise increase across all time points: baseline, 10th minute, and end of procedure (basal vs. 10 min: *p* < 0.001; basai vs. end: *p* < 0.001; 10-min vs. end: *p* = 0.040). (iii) Systolic blood pressure decreased from a baseline median of 124 mmHg to 96 mmHg at the 10th minute, followed by a partial recovery to 108 mmHg by the end of the procedure (basal vs. 10 min: *p* < 0.001; basal vs. end: *p* < 0.001; 10-min vs. end: *p* = 0.005). (iv) Diastolic blood pressure and MAP also showed significant perioperative fluctuations (all *p*-values < 0.001), with modest increases between the 10th minute and end of the procedure (diastolic BP 10-min vs. end: *p* = 0.009; MAP 10-min vs. end: *p* = 0.002). These findings indicate that both hemodynamic and respiratory parameters undergo significant changes during POEM procedures, highlighting the importance of continuous intraoperative monitoring.

Functional esophageal parameters revealed a median basal LES pressure of 23.4 mmHg (IQR: 18.0–34.0) and a basal UES pressure of 51.0 mmHg (IQR: 33.0–60.0), consistent with hypercontractile esophageal disorders. The mean incisor-to-LES distance was 40.2 cm (IQR: 38.5–41.5), which is important for intraoperative measurement and myotomy planning. The median length of hospital stay was 2 days (IQR: 1–3), suggesting favorable recovery and early discharge in most patients.

Preoperative and postoperative hematologic parameters were comparatively analyzed. There was a statistically significant increase in WBC (from 7.4 to 10.7 × 10^9^/L, *p* = 0.001) and neutrophil count (from 3.8 to 9.2 × 10^9^/L, *p* = 0.001), indicative of a physiological inflammatory response to the procedure. Hemoglobin and hematocrit levels declined slightly but significantly (*p* = 0.007 and *p* = 0.001), consistent with minor blood loss and hemodilution. Platelet count decreased from 245 to 216 × 10^9^/L (*p* = 0.008), and Red cell distribution width (RDW) also declined slightly (*p* = 0.013), although the clinical significance of this finding is uncertain. These hematologic fluctuations were transient and did not necessitate transfusion or further intervention (Table 3).

Perioperative and early postoperative complications were generally mild and self-limiting, with 27 events observed across 22 (43.1%) patients, indicating that some individuals experienced more than one adverse event during the perioperative period. These complications were identified through a combination of clinical evaluation and radiologic imaging, allowing for comprehensive assessment and timely intervention when necessary.

The most frequent early complication was subcutaneous emphysema (19.6%), followed by minor bleeding (9.8%), pneumoperitoneum (7.84%), pleural effusion (5.88%), pneumothorax (3.92%), and pneumomediastinum (3.92%). Two patients required tube thoracostomy due to pneumothorax. Only one case of major bleeding (1.96%) was observed, and it was managed conservatively. Notably, no patient developed a complication severe enough to require surgical exploration. During a median follow-up of 546 days (IQR: 374–965), no procedure-related mortality was observed. Long-term complications were rare and included symptomatic GERD in two patients (3.92%) and endoscopic esophagitis in one patient (1.96%). These findings confirm that POEM is associated with a low incidence of severe complications and favorable long-term outcomes (Table 2).

## 4. Discussion

POEM is a minimally invasive endoscopic treatment for achalasia and other esophageal motility disorders. It consists of mucosal incision, submucosal tunneling, and myotomy across the GEJ, followed by endoscopic clip closure of the mucosal entry. Emerging evidence consistently shows that POEM is best performed under general endotracheal anesthesia rather than sedation, primarily to reduce aspiration and CO_2_-insufflation–related events and to maintain a still operative field [36,59]. Accordingly, strict airway protection is essential: RSI, semi-reclined/semi-sitting intubation, and consideration of pre-procedural esophageal clearance in selected patients. Intraoperatively, pressure-controlled ventilation with adjustment of minute ventilation to maintain ETCO_2_ approximately 35 to 45 mmHg is recommended, while using low-flow CO_2_ insufflation to minimize capnomediastinum, capnoperitoneum, and subcutaneous emphysema. A rising PIP (Pmax) and abdominal distension should trigger communication with the endoscopist for immediate measures (reduced insufflation, gastric suction, brief pause), with percutaneous needle decompression reserved for persistent physiologic compromise (ETCO_2_ > 45–50 mmHg and/or Pmax > 35–38 cmH_2_O or ≥20% increase over baseline) [36,39]. Because hyperventilation alone does not correct hypercarbia once extensive subcutaneous emphysema develops, early recognition and, if necessary, subcutaneous needle venting are important. Practical steps include using a cuffed flexometallic endotracheal tube secured to the right oral commissure to facilitate endoscope passage. In our series, maintenance anesthesia with desflurane at a target MAC of 1.0 (in 50% air–oxygen) supported stable intraoperative conditions and rapid recovery, aligning our practice with current anesthetic recommendations for POEM.

While POEM is generally recognized as safe and effective, there are some patient groups for whom POEM would not be appropriate in an endoscopy unit and may require operating room conditions. Patients with complex anatomy or previous surgery in the upper gastrointestinal tract may have adhesions or altered anatomy that make it difficult to safely perform POEM in the endoscopy unit. In such cases, the procedure may be better suited for an operating room with more space and equipment. Patients with significant comorbidities, such as severe cardiopulmonary disease or bleeding disorders, may benefit from the controlled environment of an or where additional medical support and resources are readily available. In our hospital, POEM procedures are safely performed in the operating room with multidisciplinary support, although the endoscopy unit has an anesthesia device and equipment to respond to all emergencies.

Perioperative pulmonary aspiration is a rare but potentially serious cause of anesthesia-related morbidity and mortality and is one of the leading anesthetic challenges. It is also among the primary causes of death from pulmonary complications. The most important factor in the induction of anesthesia in patients with achalasia is the prevention of aspiration. Aspiration occurs in approximately 3/10,000 anesthesia procedures, with a higher frequency in special patient groups and emergencies [36,60,61]. However, patients with achalasia have a higher risk of aspiration than the general population, as the presence of food in the esophagus cannot be confirmed without prophylactic EGD. Therefore, in most cases, RSI is mandatory [36,37,59,62]. Induction and intubation in a semi-sitting position may be another option. The risk of aspiration of esophageal and gastric contents can be minimized by using short-onset hypnotics and neuromuscular blocking agents and by minimizing positive pressure ventilation with the Sellick maneuver and face mask. When rocuronium 1.2 mg/kg is used with propofol 2–3 mg/kg, RSI conditions can be achieved [63,64,65]. Similarly to the literature, in the present study, we performed intubations in a semi-sitting position with RSI using propofol and rocuronium, applying cricoid pressure to protect our patients from this dreaded complication. After intubation, the endotracheal tube was fixed on the right side of the mouth to facilitate endoscope access. The patients were intubated with a spiral tube so that the endotracheal tube would not fold in the mouth. However, the level of the tube should be checked frequently because the position of the intubation tube may change as the endoscope is constantly moved in the oral cavity.

During the procedure, CO_2_ insufflation is mandatory throughout the entire procedure to improve the field of view and to evaluate the passage. CO_2_ insufflation during POEM may lead to systemic CO_2_ uptake and increased intra-abdominal pressure. The possibility of CO_2_ leakage into various tissue spaces should be kept in mind [37], and pneumomediastinum, pneumoperitoneum, and subcutaneous and submucosal emphysema are possible. Since the likelihood of these complications is frequent, it is imperative to be prepared to manage complications [37]. PaCO_2_ is usually 4 to 5 mmHg higher than ETCO_2_. ETCO_2_ is most widely used as a non-invasive alternative to PaCO_2_ in assessing the adequacy of ventilation during laparoscopic surgery. There may be significant differences between ETCO2 and PaCO_2_ due to V/Q mismatch. Therefore, direct estimation of PaCO2 by arterial blood gas analysis may be required to detect hypercarbia. Therefore, artery cannulation for continuous blood pressure monitoring and frequent arterial blood gas analysis should be considered in patients with preoperative cardiopulmonary disease and in cases of intraoperative hypoxemia, high airway pressures, or high ETCO_2_ [66]. In the present study, radial artery cannulation was performed in 5 patients, either with cardiovascular disease or who developed significant CO_2_ leakage–related complications during the procedure, requiring close monitoring. Increases in ETCO_2_ were closely monitored with a capnograph, and appropriate increases in minute ventilation were made to keep it below 45 mmHg.

POEM-related complications can be categorized into early and late complications. The most frequent early complications include subcutaneous emphysema (4.9–7.5%), pneumoperitoneum (6.8–15.5%), pleural effusion (0.9–1.2%), pneumothorax (1.2%), pneumomediastinum (1.1–1.8%), mucosal injury (1.5–4.8%), major or minor bleeding (0.2–0.6%), and esophageal perforation (0.2–0.3%) [41,67,68]. The late complications are predominantly symptomatic GERD (8.5–19%), esophagitis (13–29.4%), and more rarely peptic strictures and Barrett’s esophagus [67,69,70,71,72,73]. Most of the early complications associated with POEM are related to CO_2_ insufflation, and gas-related complications may be observed as a result of inadvertent entry of gas into the surrounding tissues [36,59]. Since great effort is required to detect, reduce and treat these complications, anesthesiologists are concerned about these risks [36]. In the present study, a total of 27 complications were observed in 22 patients, of which 18 were secondary to CO_2_ leakage. Subcutaneous emphysema was seen in 10 patients, pneumoperitoneum in 4 patients, pneumomediastinum in 2 patients, and pneumothorax in 2 patients. In case of a significant increase in ETCO_2_, the endoscopist should be informed, and CO_2_ insufflation should be minimized as much as possible.

As can be understood from the low doses of opioids we use in induction, this procedure is not stimulating in itself. In these procedures, post-procedural pain is generally not an issue [37]. It has been reported that a multimodal approach including a combination of acetaminophen, non-steroidal anti-inflammatory drugs, and moderately effective opioids will be sufficient in the pain management of these patients [74]. We routinely administered tramadol and paracetamol analgesics to our patients to prevent postoperative pain. Patients are usually kept in the intensive care unit overnight to monitor for any complications. We extubated our patients and followed them in the intensive care unit for one day for close follow-up. They were discharged after 2 days of follow-up in the ward. The 3 patients who developed pneumothorax and underwent chest tube insertion were discharged on the 10th day after 3 days in the intensive care unit and then in the ward.

Routine preoperative endoscopy, although considered the gold standard for assessing esophageal clearance, was not included in our protocol due to its invasive nature and practical limitations. This absence may represent a limitation in terms of aspiration risk assessment, as it introduces uncertainty regarding residual esophageal contents [37]. While prolonged fasting, RSI, and cricoid pressure are commonly used protective strategies, they do not confirm complete esophageal emptying [37]. In this context, point-of-care ultrasonography (POCUS) has emerged as a non-invasive and real-time imaging modality with growing clinical interest [75]. Recent studies have demonstrated that POCUS can accurately estimate gastric residual volume, esophagial dilatation and identify the presence of solid or liquid content in fasting patients [76,77]. Although the evidence supporting its use for direct esophageal assessment remains limited, early data suggest that POCUS could contribute to aspiration risk stratification in selected patients [78]. Therefore, incorporating POCUS into preoperative workflows may enhance both patient safety and procedural efficiency in POEM.

Another important aspect worth discussing is whether routine ICU monitoring is necessary for all patients undergoing POEM. Although all patients in our study were monitored in the ICU for 24 h postoperatively, we acknowledge that this approach may not be applicable across all healthcare systems, particularly those with limited ICU availability. Our approach is also supported by a study that have emphasized the role of close postoperative monitoring, especially during the early phase of implementing a new protocol, in order not to overlook early postoperative complications [59]. In patients without comorbidities or procedure-related complications, routine ICU admission may lead to unnecessary use of critical care resources. Therefore, individualized risk stratification should guide postoperative care planning. For low-risk patients, implementation of enhanced recovery after surgery protocols may promote earlier discharge and minimize the need for prolonged ICU monitoring, thereby optimizing resource utilization without compromising patient safety [79,80].

### Limitations

This study has several limitations. First, it was conducted at a single tertiary care center with a relatively small sample size, which may limit the statistical power and generalizability of the findings. Second, its retrospective design introduces potential biases, including reliance on the accuracy and completeness of recorded data, which may have led to underreporting of intraoperative events. Finally, long-term follow-up beyond the reported median period was not available for all patients. Future prospective, multicenter studies with larger cohorts are warranted to validate these findings.

## 5. Conclusions

This study demonstrates that POEM can be performed safely and effectively under general anesthesia when supported by structured multidisciplinary collaboration and vigilant intraoperative monitoring. Although transient physiological changes such as CO_2_ accumulation and blood pressure fluctuations are expected due to insufflation and procedural complexity, they remained within manageable limits in our study. No procedure-related mortality occurred, and the rate of serious complications was low. These outcomes emphasize the importance of tailored anesthetic protocols—including aspiration prevention strategies and proactive ventilation management—to ensure patient safety. Ultimately, the success of POEM depends not only on technical expertise but also on the integration of anesthetic precision, endoscopic skill, and coordinated perioperative care.

## Figures and Tables

**Table 1:** Demographic and clinical profile of patients undergoing POEM.

Categorical Variables	Results
Gender (%)	
Male	24 (47.1)
Female	27 (52.9)
Blood Groups	
0 Rh(+)	23 (45.1)
A Rh(+)	12 (23.5)
B Rh(+)	14 (27.5)
AB Rh(+)	2 (3.9)
Comorbidities (%)	
DM	6 (11.8)
HT	16 (31.4)
ASA Scores (%)	
ASA II	33 (64.7)
ASA III	18 (35.3)
Post POEM early complication (%; *n* = 22 patients)	
Major bleeding	1 (1.96)
Minor bleeding	5 (9.8)
Subcutaneous emphysema	10 (19.6)
Pneumothorax	2 (3.92)
Pleural effusion	3 (5.88)
Pneumoperitoneum	4 (7.84)
Pneumomediastinum	2 (3.92)
Post POEM long term complication (%)	
Symptomatic GERD	2 (3.92)
Esophagitis	1 (1.96)

ASA: American Society of Anesthesiologists, DM: Diabetes Mellitus, HT: Hypertension, POEM: Peroral Endoscopic Myotomy, Rh: Rhesus.

**Table 2:** Intraoperative hemodynamic and physiologic parameters of patients undergoing POEM.

Continuous Variables	Median	95% CI	IQR
Age	48	46–55	40–63
Height	164	162–168	160–173
Weight	55	53–59	50–63
SpO_2_ (%)			
^1^ Basal	95	95–96	94–96
^2^ 10 min	98	98–99	95–99
^3^ End of the procedure	98	98–99	95–99
*p*-value	<0.001 [1 vs. 2 (<0.001); 1 vs. 3 (<0.001)]		
ETCO_2_ (mmHg)			
^1^ Basal	34	34–36	32–38
^2^ 10 min	46	45–48	41–50
^3^ End	49	48–51	45–52
*p*-value	<0.001 [1 vs. 2 (<0.001); 1 vs. 3 (<0.001); 2 vs. 3 (0.040)]		
Sistolic pressure (mmHg)			
^1^ Basal	124	119–128	115–133
^2^ 10 min	96	92–107	88–109
^3^ End of the procedure	108	104–115	97–119
*p*-value	<0.001 [1 vs. 2 (<0.001); 1 vs. 3 (<0.001); 2 vs. 3 (0.005)]		
Diastolic pressure (mmHg)			
^1^ Basal	72	70–76	63–79
^2^ 10 min	58	58–61	54–62
^3^ End of the procedure	60	58–65	55–68
*p*-value	<0.001 [1 vs. 2 (<0.001); 1 vs. 3 (<0.001); 2 vs. 3 (0.009)]		
MAP (mmHg)			
^1^ Basal	88	85–93	81–96
^2^ 10 min	71	69–75	65–78
^3^ End of the procedure	76	73–83	70–86
*p*-value	<0.001 [1 vs. 2 (<0.001); 1 vs. 3 (<0.001); 2 vs. 3 (0.002)]		
PIP (mmHg)	25	24–26	25–26
Anesthesia Duration	180	180–185	165–185
Basal LES (mmHg)	23.4	21.8–27.0	18.0–34.0
Basal UES (mmHg)	51.0	41.6–54.1	33.0–60.0
Incisor-to-LES distance	40.2	39.7–41.0	38.5–41.5
Length of stay (days)	2	2–3	1–3

SpO_2_: oxygen saturation, MAP: mean arterial pressure, PIP: peak inspiratory pressure, LES: lower esophageal sphincter, UES: upper esophageal sphincter, ETCO_2_: end-tidal carbon dioxide. In Table 2, numerical indicators (1, 2, and 3) were used in the *p*-value rows due to limited space. These numbers correspond to comparisons across three time points: 1: baseline measurement, 2: measurement at the 10th minute, and 3: measurement at the end of the procedure.

**Table 3:** Comparison of preoperative and postoperative hematologic parameters in POEM patients.

Variables	PrePOEM			PostPOEM			*p*
	Median	95% CI	IQR	Median	95% CI	IQR	
Hemoglobin	12.5	12.1–13.3	11.2–14.0	12.3	11.8–13.2	11.3–13.4	0.007
Hematocrit	38.9	36.6–40.2	35.6–42.1	37.5	35.8–39.1	35.1–40.4	0.001
WBC	7.4	6.8–7.9	6.26–9.30	10.7	9.7–11.5	7.9–11.7	0.001
Neutrophils	3.8	3.7–4.3	3.3–4.8	9.2	8.6–9.8	7.2–10.7	0.001
RDW	40.8	40.1–41.9	39.6–42.5	40.6	40.1–41.8	39.1–42.3	0.013
Platelet	245	206–266	195–287	216	201–240	180–285	0.008

CI: Confidence Interval, IQR: Interquartile Range (Q1–Q3), RDW: Red Cell Distribution Width, WBC: White Blood Cell.

## Data Availability

The data presented in this study are available upon request from the corresponding author. The ethics committee requests a confidentiality letter for the management of this study’s data due to the inclusion of personal and clinical patient data.
